# Dual pH- and GSH-Responsive Degradable PEGylated Graphene Quantum Dot-Based Nanoparticles for Enhanced HER2-Positive Breast Cancer Therapy

**DOI:** 10.3390/nano10010091

**Published:** 2020-01-02

**Authors:** Na Re Ko, Se Young Van, Sung Hwa Hong, Seog-Young Kim, Miran Kim, Jae Seo Lee, Sang Ju Lee, Yong-kyu Lee, Il Keun Kwon, Seung Jun Oh

**Affiliations:** 1 Biomedical Research Center, Asan Institute for Life Sciences, 88 Olympic-ro 43-gil, Songpa-gu, Seoul 05505, Korea; nare.ko@amc.seoul.kr (N.R.K.); sey.van88@gmail.com (S.Y.V.); 2 Department of Nuclear Medicine, Asan Medical Center, University of Ulsan College of Medicine, 88 Olympic-ro 43-gil, Songpa-gu, Seoul 05505, Korea; ranaranmi@naver.com (M.K.); atlas425@amc.seoul.kr (S.J.L.); 3 Department of Chemical Engineering and Applied Chemistry, University of Toronto, 200 College Street, Toronto, ON M5S 3E5, Canada; sunghwa.hong@mail.utoronto.ca; 4 Department of Convergence Medicine, University of Ulsan College of Medicine, 88 Olympic-ro 43-gil, Songpa-gu, Seoul 05505, Korea; ksy0222@gmail.com; 5 Department of Dental Materials, School of Dentistry, Kyung Hee University, 26 Kyungheedae-ro, Dongdaemun-gu, Seoul 02447, Korea; leejs8433214@naver.com (J.S.L.); kwoni@khu.ac.kr (I.K.K.); 6 Department of Chemical & Biological Engineering, Korea National University of Transportation, 50 Daehak-ro, Chungcheongbuk-do, Cheongju 27469, Korea; leeyk@ut.ac.kr

**Keywords:** stimuli-responsive degradation system, glutathione, breast cancer, Herceptin, active targeting, PEGylation

## Abstract

Dual stimuli-responsive degradable carbon-based nanoparticles (DS-CNPs) conjugated with Herceptin (HER) and polyethylene glycol (PEG) have been designed for the treatment of HER2-positive breast cancer. Each component has been linked through disulfide linkages that are sensitive to glutathione in a cancer microenvironment. β-cyclodextrin (β-CD) on the surface of DS-CNPs formed an inclusion complex (DL-CNPs) with doxorubicin (DOX) at a high loading capacity of 5.3 ± 0.4%. In response to a high level of glutathione (GSH) and low pH in a tumor environment, DL-CNPs were rapidly degraded and released DOX in a controlled manner via disruption of host–guest inclusion. These novel DL-CNPs exhibited high cellular uptake with low toxicity, which induced the efficient inhibition of antitumor activity both in vitro and in vivo. Cell viability, confocal laser scanning microscopy, and animal studies indicate that DL-CNPs are a great platform with a synergistically enhanced antitumor effect from the dual delivery of HER and DOX in DL-CNPs.

## 1. Introduction

Recently, stimuli-responsive degradation (SRD)-induced drug delivery systems (DDS) have received much attention in the field of nanomedicine. SRD enables nanotechnology-based DDS to achieve both selective uptake at target disease sites and controlled drug release with low side effects. SRD-nanomedicine platforms can be engineered with dynamic covalent linkages that can be cleaved in response to external stimulus such as glutathione (L-γ-glutamyl-L-cysteinyl-glycine; GSH), pH, enzymes, temperature, and glucose. These environmental triggers are considered safe and efficient as stimuli-responsive nanocarriers for cancerous diseases [1,2,3]. In particular, GSH is an antioxidant that maintains the intracellular redox balance, and elevated GSH levels are associated with chemoresistance of neoplastic tissue [4,5,6]. GSH is distributed at different concentrations within the tumor milieu such as in the intracellular compartments of a tumor cell at 2–10 mM, and in the extracellular compartments of a tumor cell at <20 μM [7,8]. Disulfide linkages are cleavable by a reductive environment or a disulfide-thiol exchange reaction [9,10,11], and upon exposure to GSH, cleavage of the disulfides accelerates destabilization of disulfide-labeled drug carriers and controls the release of encapsulated anticancer therapeutics within the carriers [12]. In addition, the pH difference between tumor/inflamed regions (pH 5.4–7.1) and normal tissue/blood stream (pH~7.4) enables cancer cells to promote cell–matrix remodeling, increase the activity of acid-activated proteases, and progress tumor growth [13,14]. Such a difference in the tumor microenvironment is useful tools for designing cancer-specific multifunctional biomimetic nanoparticles. Various pH-responsive degradable drug carriers possess acid-labile cleavable linkages such as ester [15,16], amide [17,18], and ketal/acetal groups [19,20].

Carbon-based nanoparticles (CNPs) including graphenes, carbon nanotubes, and fullerenes, have become attractive materials in the field of biomedical applications [21]. Due to their good biocompatibility, and structural, physical, and chemical properties, CNPs have been investigated for their use as biosensors, drug delivery carriers, and in imaging probes [22]. Surface-functionalized CNPs enable modifications and conjugations with chemical/biomolecular agents such as drugs, antibodies, and immunoglobulin [23,24,25]. In this respect, a Herceptin (HER)-conjugated GSH-responsive degradable graphene quantum dot (GQD)-based nanomaterial has been developed to target HER2-positive breast cancer [26]. HER2 (Human Epidermal growth factor Receptor 2) is a protein on the surface of breast cells made by HER2 genes. Overexpressed HER2 genes produce up to 100-fold of HER2 proteins and also cause uncontrolled cell growth, which leads to HER2-positive breast cancer [27]. HER is a FDA-approved monoclonal antibody that binds to HER2 to inhibit intracellular signaling and induce the immune response of HER2-overexpressed breast cancer [28,29]. However, due to the low antitumor efficiency of HER, chemotherapy is required to enhance anticancer activity for clinical use [30]. Polyethylene glycol (PEG)-labeled GQDs enable a prolonged blood circulation time and have a stealth effect on the host immune system. In addition, disulfides at conjugate junctions have been cleaved in response to the high GSH level in breast cancer cells and enable the controlled release of biomolecular- (HER) and chemo-anticancer therapeutics (doxorubicin; DOX). However, such multiple stimuli-responsive graphenes have seldom been reported [31] and dual redox- and pH-responsive GQD-based nanocarriers for breast cancer therapy have not been investigated to date.

**Scheme 1:**
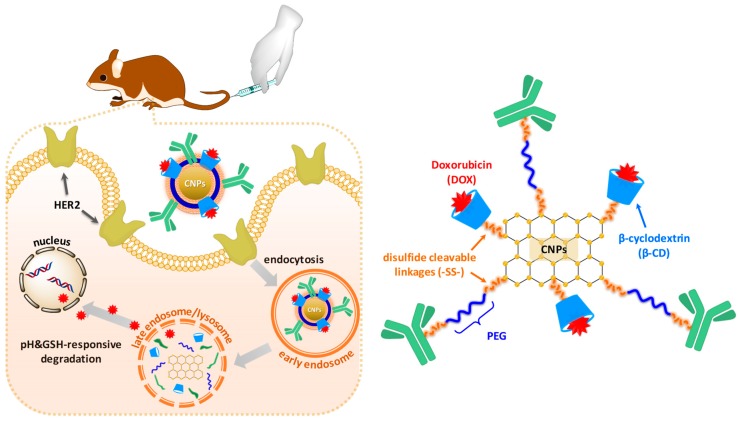
Illustration of cellular uptake and drug release of DOX-loaded DS-CNPs (DL-CNPs) in HER2-positive breast cancer cells.

In this study, novel dual stimuli-responsive graphene quantum dot-based multi-functional carbon nanoparticles (DS-CNPs) were developed for use in breast cancer therapy (Scheme 1). The particles were modified with both PEG and HER, and they have pH-dependent ester/amide bonds and GSH-sensitive disulfide linkages at multiple locations. A drug pocket β-cyclodextrin (βCD) with a hydrophobic cavity and hydrophilic surface enhances the solubility of the hydrophobic anticancer drug, DOX, via host–guest chemistry. In addition, βCD is known to release possessing drugs in response to multiple external stimuli including temperature, changes in pH or redox, light, and competitive binding [32,33,34]. These engineered drug carriers allow not only active targeting, but they also enhance the anticancer effect with the controlled release of HER therapeutics. When DOX-loaded DS-CNPs (DL-CNPs) larger than 200 nm are intravenously injected, they accumulate in the breast cancer region via an enhanced permeability and retention effect. After endocytosis of DL-CNPs, both HER and DOX are released upon exposure to low pH and a high level of GSH. HER not only specifically guides the DL-CNPs to HER2-positive breast cancer, but it also suppresses tumor growth [35]. The released DOX then penetrates the cell nucleus and causes cell death by damaging the DNA [36]. Combined, the cell viability, confocal laser scanning microscopy (CLSM), and in vivo studies suggest that DL-CNPs have great potential for use in the treatment of HER2-positive breast cancer.

## 2. Materials and Methods

### 2.1. Materials

Amine-functionalized CNPs were synthesized by the pyrolysis of L-glutamic acid (the detailed procedure is described in our previous report [25]). 3,3′-Dithiodipropionic acid (SS-COOH, 99%), polyethylene glycol (PEG, MW = 3000 g/mol), 1-ethyl-3-(3-dimethylaminopropyl)-carbodiimide (EDC, >97%), 4-dimethylaminopyridine (DMAP, >98%), N-hydroxysuccinimide (NHS, 98%), and doxorubicin hydrochloride (DOX, -NH_3_^+^Cl^−^ salt form, >98%) were obtained from Aldrich (St. Louis, MO, USA), and βCD (>98%) obtained from TCI (Tokyo, Japan) were purchased and used as received. HER was purchased from Roche (Basel, Switzerland), and Roswell Park Memorial Institute (RPMI) 1640 media, Dulbecco’s phosphate-buffered saline, trypsin-EDTA, and penicillin-streptomycin were purchased from Thermo Fisher Scientific (Waltham, MA, USA). Fetal bovine serum (FBS), CCK-8 reagent (product # CK04-13), and lysosomal staining reagent (product # ab176829) were purchased from GIBCO BRL (Grand Island, NY, USA), Dojindo (Kumamoto, Japan), and Abcam (Cambridge, MA, USA), respectively.

### 2.2. Instrumentation

The synthesis of βCD-SS-COOH, PEG-(SS-COOH)_2_ was confirmed by Fourier transform nuclear magnetic resonance spectrometer (NMR, AVANCE III HD400, Bruker, Rheinstetten, Germany); and βCD-SS-CNPs and DS-CNPs were characterized by Fourier transform infrared (FTIR) spectroscopy (Thermo Scientific Nicolet 380 spectrometer, Waltham, MA, USA) using the KBr Pellet technique with scanning in the wavenumber range 400–4000 cm^−1^. The conjugation of HER with PEG-(SS-COOH)_2_ was confirmed by x-ray photoelectron spectroscopy (XPS, Thermo Fisher, K-alpha, East Grinstead, UK). UV/Vis spectra were recorded using a PerkinElmer Lambda 650S UV/Vis spectrometer (Waltham, MA, USA). Thermal gravimetric analysis (TGA) was conducted to confirm the synthesis of DS-CNPs using TA Instruments 2960 SDT V3.0F (New Castle, DE, USA, 10 °C/min, 10–800 °C, at atmospheric pressure under nitrogen flow). The change in particle size during synthesis was evaluated by dynamic light scattering (DLS) using a particle size analyzer (ELSZ-1000, Otsuka Electronics Co. Ltd., Osaka, Japan).

### 2.3. Carboxylation of βCD and Conjugation with CNPs (βCD-SS-CNPs)

In a solution of SS-COOH (5.56 g, 26.4 mmol) dissolved in 20 mL DMF, DMAP (108 mg, 0.88 mmol) was added and stirred at 50 °C for 10 min. βCD (10 g, 8.8 mmol) was dissolved in 10 mL DMF and added to a mixture of SS-COOH/DMAP. Finally, EDC (1.37 g, 8.8 mmol) dissolved in 10 mL DMF was added, and the reaction mixture was stirred at 50 °C for 24 h. The resulting βCD-SS-COOH was obtained by dialysis (MWCO = 1000 g/mol) against distilled water (DW) for two days and lyophilization. The purified and dried βCD-SS-COOH (500 mg) was dissolved in the presence of DMAP (27 mg, 0.22 mmol) in 30 mL PBS and added drop-wise into a solution of 35 mg CNPs and EDC (64 mg, 0.41 mmol) in PBS (5 mL). The reaction mixture was stirred in the dark at room temperature (RT) for two days. The solution was filtered, and the filtrates were dialyzed using dialysis tubing (MWCO = 1000 g/mol) against DW for two days to remove residual by-products and then lyophilized.

### 2.4. Carboxylation of PEG and Conjugation with HER (SS-PEG-SS-HER)

SS-PEG-SS-HER was synthesized by the click reaction (the detailed procedure is described in our previous report) [26]. In brief, two hydroxyl end groups of PEG were substituted by SS-COOH, which resulted in PEG-(SS-COOH)_2_. SS-PEG-SS-HER was then synthesized by an EDC coupling reaction between PEG-(SS-COOH)_2_ and HER in the presence of DMAP. The purified SS-PEG-SS-HER was then lyophilized and stored at 4 °C until further use.

### 2.5. Synthesis of Dual Stimuli-Responsive Degradable CNPs (DS-CNPs)

SS-PEG-SS-HER (500 mg, 0.0034 mmol) was dissolved in 30 mL PBS in the presence of DMAP (212 mg, 1.7 mmol). EDC (2.7 g, 17.8 mmol) and NHS (2.0 g, 17.4 mmol) were then added and stirred for 10 min, and βCD-SS-CNPs was added in a solution and stirred at room temperature (RT) for 24 h. The resulting mixture was filtered, and the filtrates were then dialyzed using dialysis tubing (MWCO = 1000 g/mol) against DW for two days. DS-CNPs were lyophilized and stored at 4 °C until further use.

### 2.6. Preparation DOX-Loaded DS-CNPs (DL-CNPs)

DS-CNPs (20 mg) were dissolved in 2 mL DMSO in the presence of 2 mg DOX and Et_3_N (3 molar equivalents to DOX) and stirred at RT for 24 h. PBS (58 mL) was then added drop-wise into the solution and stirred at RT for 24 h. The mixture was transferred into a dialysis membrane (MWCO = 1000 g/mol) and dialyzed against 1 L DW for 24 h and PBS buffered for 24 h. To determine the loading capacity (LC) and encapsulation efficiency (EE) of DOX in DL-CNPs, an aliquot of the DL-CNPs was mixed with DMF (1:3 v/v). Then, the amount of encapsulated DOX was measured using the Beer–Lambert equation with the absorbance at λ_max_ = 498 nm and the previously reported extinction coefficient (ε = 12,800 M^−1^ cm^−1^) [25]. The LC and EE of DL-CNPs were calculated as follows;

$$\mathrm{LC}\;=\;\frac{\mathrm{weight}\;\mathrm{of}\;\mathrm{DOX}\;\mathrm{in}\;\mathrm{DL}-\mathrm{NPs}}{\mathrm{total}\;\mathrm{weight}\;\mathrm{of}\;\mathrm{DL}-\mathrm{NPs}}$$

$$\mathrm{EE}\;=\;\frac{\mathrm{weight}\;\mathrm{of}\;\mathrm{DOX}\;\mathrm{in}\;\mathrm{DL}-\mathrm{NPs}}{\mathrm{initial}\;\mathrm{amount}\;\mathrm{of}\;\mathrm{DOX}\;\mathrm{added}}$$

### 2.7. pH and GSH-Triggered Release of DOX from DL-CNPs

DL-GQD solution (3 mL) was placed in dialysis tubing (MWCO = 1000 g/mol) and then dialyzed against 50 mL PBS buffer under different conditions: 10 mM GSH at pH 5.5; 10 mM GSH at pH 7.4; 2 μM GSH at pH 5.5; and 2 μM GSH at pH 7.4 as the control. All experiments were conducted in the dark to prevent DOX from decomposing. The absorbance of DOX in outer water was taken periodically for 48 h using a UV/Vis spectrometer at λ_ex_ = 498 nm.

### 2.8. Cell Culture

Both cell types were cultured in RPMI 1640 containing L-glutamine, 25 mM HEPES, 10% fetal bovine serum, and 1% antibiotics (50 units/mL penicillin and 50 units/mL streptomycin). Cells were incubated at 37 °C in a humidified atmosphere containing 5% CO_2_ until appropriate confluency, and fresh media were provided every 3–4 days.

### 2.9. Protein Preparation and Immunoblot Analysis

SK-BR-3 and MDA-MB-231 cells were harvested with 1X PBS buffer and suspended in lysis buffer (Cell Signaling Technology, Beverly, MA, USA), then boiled for 5 min. Protein concentrations were measured with the BCA protein assay (Pierce, Rockford, IL, USA). The samples were diluted with 1X lysis buffer and an equal amount of protein was loaded on 10% SDS-polyacrylamide gel. SDS-polyacrylamide gel electrophoresis (PAGE) analysis was performed using a Bio-rad gel apparatus. Proteins were separated by SDS-PAGE, transferred to nitrocellulose membranes, and blocked with 5% skim milk in TBS-Tween 20 (0.05%) for 30 min. The membrane was incubated with primary antibodies against HER/ErbB2 (Cell Signaling Technology, Danvers, MA, USA) and β-actin (Sigma, St. Louis, MO, USA) overnight at 4 °C. Horseradish peroxidase-conjugated anti rabbit IgG used as the secondary antibody (Jackson immunoresearch, West Grove, PA, USA) was incubated with the membrane for 1 h at RT. Protein quantification analysis was performed using the CS Analyzer 4 program (ATTO corporation, Tokyo, Japan).

### 2.10. Cell Viability Using the CCK-8 Assay

SK-BR-3 and MDA-MB-231 cells were seeded at a density of 5 × 10^3^ cells per well into a 96-well plate (n = 3) in 100 μL of RPMI 1640 media, and were then allowed to adhere on the substrate for 3 h. Cells were then treated with DS-CNPs (0–500 μg/mL), DL-CNPs (0–40 μg/mL), and equivalent amounts of free DOX (calculated from the LC) at the same concentration ranges of 0 to 3.67 μM for 48 h. The culture media were then aspirated, and 100 μL of fresh media with 10 μL of CCK-8 reagent was added in every well and then incubated for 2 h at 37 °C. The absorbance value was measured at 450 nm using a microplate spectrophotometer (Infinite 200 Pro, Tecan, Switzerland).

### 2.11. Confocal Laser Scanning Microscopy (CLSM)

SK-BR-3 and MDA-MB-231 cells were plated on a glass bottom cell culture confocal dish (Φ 20 mm) at a density of 1 × 10^5^ cells per well and then incubated for 24 h under 37 °C and 5% CO_2_ in a humidified incubator. At 24 h, cells were treated with both DL-CNPs (43.48 μg/mL) and free DOX (4.0 μM, which is an equivalent amount to the DL-CNPs) in a time-dependent manner (3 h and 8 h). Lysosomal staining reagent diluted, according to the manufacturer’s protocols, was incubated with cells for 30 min before observation to clarify the cytoplasm area. DOX and lysosomal staining reagent were observed at a wavelength of excitation/emission of 488/589 nm and 633/665 nm, respectively, using a confocal laser scanning microscope (LSM 880, ZEISS, Oberkochen, Germany).

Fluorescence images were quantified using ImageJ software (NIH). A total of 30 cells were randomly selected from each experimental group for image analysis (an average of 10 cells per image). The DOX uptake within the nucleus was analyzed based on the fluorescence intensity ratio of the nucleus to the whole cell area. The mean gray value of the nucleus divided by that of the whole cell area was presented as a percentage. A statistical analysis of the data was conducted using GraphPad Prism 5 (GraphPad Software, San Diego, CA, USA). The student *t*-test was applied to compare two time points of the DL-CNP groups in each cell, and the statistical significance was denoted as * (*p* < 0.05), ** (*p* < 0.01), or *** (*p* < 0.001).

### 2.12. Animal Studies

The research protocol was approved by the Institutional Animal Care and Use Committee of the Asan Institute for Life Science (registration no. 2018-13-066). Athymic nude mice (Balb/c-nu, female, 5–6 weeks old, 20–22 g) and NSG mice (female, 5–6 weeks old, 20 g) were purchased from the Japan Shizuoka Laboratory Center. Mice were maintained in accordance with the Institutional Animal Care and Use Committee guidelines of the Asan Institute for Life Science. Mice were inoculated with exponentially growing human breast cancer cells (MDA-MB-231 or SK-BR-3, 1:1 ratio of plain media and Matrigel (Corning, 354234, 1 × 10^7^/total 0.2 mL) into the flank subcutaneously. Over the following 14 to 25 days, tumors were allowed to reach a volume of 70 to 100 mm^3^, and the tumor volume was calculated by the length × width × height × π/6. Mice were randomly allocated to the PBS, free DOX, or DL-CNPs treatment group (n ≥ 7 per group), and injected intravenously with vehicles or drugs bearing the xenograft once a week until the end point on day 27 to 38. Immunohistochemical analysis was also carried out with sectioned samples using Ki-67 (cat. No. M7240, Agilent Dako).

## 3. Results and Discussions

### 3.1. Synthesis and Characterization of DS-CNPs

**Scheme 2:**
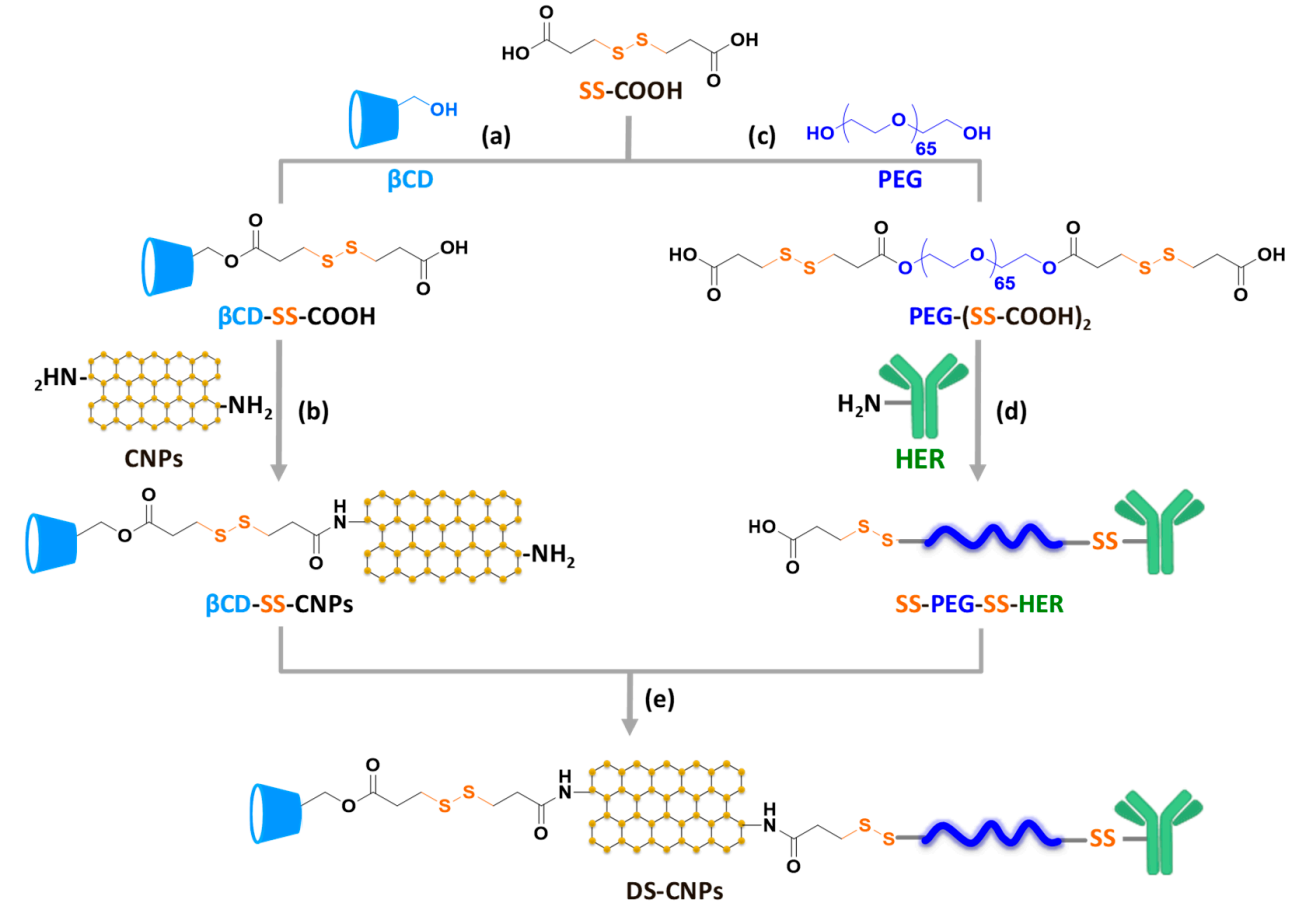
Synthetic scheme of dual stimuli-responsive degradable carbon-based nanoparticles (DS-CNPs): carboxylation of β-cyclodextrin (βCD) (**a**), conjugation of carbon-based nanoparticles (CNPs) (**b**), carboxylation of polyethylene glycol (PEG) (**c**), conjugation of Herceptin (HER) (**d**), and coupling reaction used to form DS-CNPs (**e**) via EDC/DMAP coupling reaction.

Scheme 2 illustrates the synthetic approach used to prepare dual stimuli-responsive degradable DS-CNPs consisting of β-CD and HER with multiple disulfide linkages.

**Figure 1:**
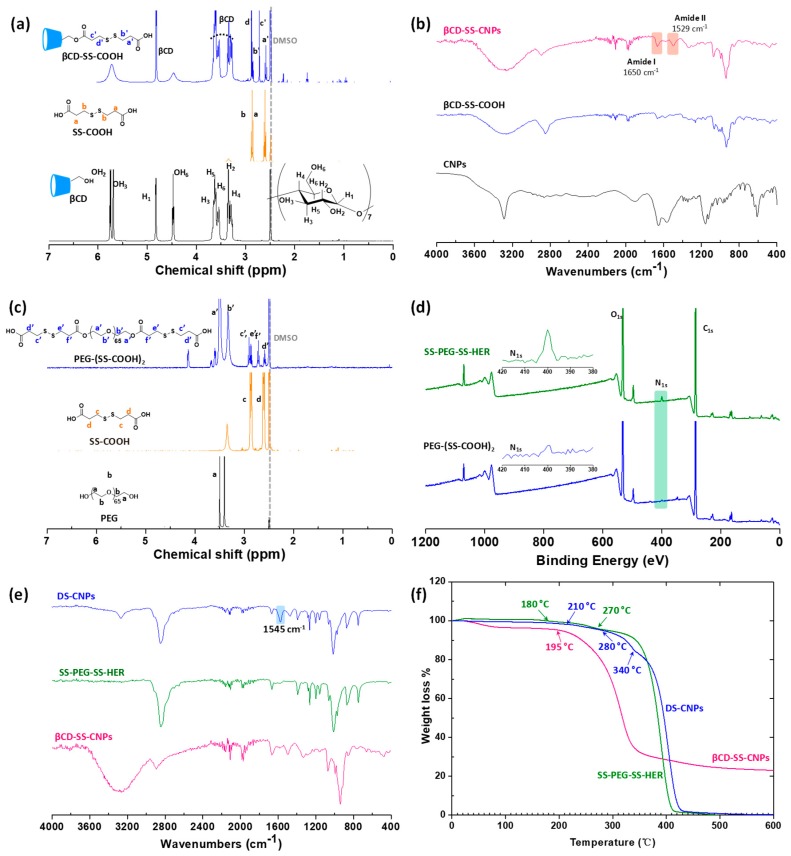
(**a**) Synthesis of βCD-SS-COOH confirmed by ^1^H-NMR, (**b**) synthesis of βCD-SS-CNPs confirmed by FTIR, (**c**) synthesis of PEG-(SS-COOH)_2_ confirmed by ^1^H-NMR, (**d**) synthesis of SS-PEG-SS-HER confirmed by XPS, synthesis of DS-CNPs confirmed by FTIR (**e**) and TGA (**f**).

**Figure 2:**
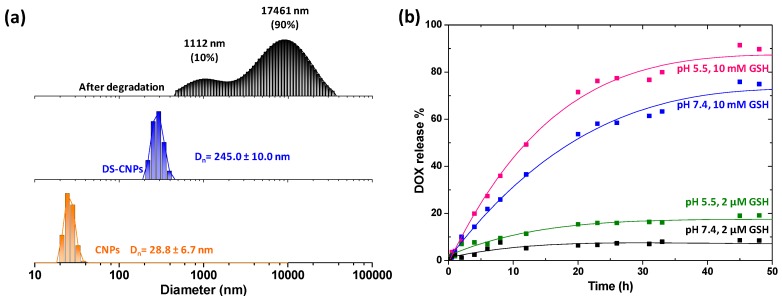
(**a**) DLS diagrams of CNP, DS-CNPs, and degraded DL-CNPs, (**b**) in vitro release profile of DOX from DL-CNPs under various conditions: 10 mM GSH at pH 5.5, 10 mM GSH at pH 7.4, 2 μM GSH at pH 5.5, and 2 μM GSH at pH 7.4 as the control.

SS-COOH was labeled with βCD and PEG separately via a facile DCC/DMAP coupling reaction to produce βCD-SS-COOH and PEG-(SS-COOH)_2_. Synthesis of βCD-SS-COOH and PEG-(SS-COOH)_2_ was clearly determined by ^1^H-NMR (Figure 1a,c). Carboxylic acid groups of βCD-SS-COOH were then covalently attached on the amine-functionalized CNPs to introduce pH-responsive amide bonds between βCD and CNPs, and the formation of amide groups was confirmed by FTIR (Figure 1b). A carboxylic acid group of PEG-(SS-COOH)_2_ was utilized to conjugate with the amine groups on HER. The increase in N content determined by XPS indicated the formation of SS-PEG-SS-HER (Figure 1d). Amine groups on the surface of partially modified βCD-SS-CNPs were then used to synthesize DS-CNPs via a coupling reaction with the remaining carboxylic acid of SS-PEG-SS-HER. The successful synthesis of DS-CNPs was determined by both the changes in the chemical bonds and the thermal properties. In Figure 1e, the appearance of a new peak at 1545 cm^−1^ showed the presence of amide bonds between βCD-SS-CNPs and SS-PEG-SS-HER. TGA analysis showed a single transition in weight loss at 195 °C for βCD-SS-CNPs and two temperature transitions at 180 °C and 270 °C for SS-PEG-SS-HER. After conjugation, DS-CNPs underwent three temperature transitions at 210 °C, 280 °C, and 340 °C, which indicates that the DS-CNPs possessed both βCD-SS-CNPs and SS-PEG-SS-HER components. After surface modification, the particle sizes of DS-CNPs significantly increased from 28.8 ± 6.7 nm to 245.0 ± 10.0 nm (Figure 2a).

### 3.2. Loading and External Stimuli-Trigger Release of DOX

DOX is an anticancer drug with poor water solubility. In the design of DS-CNPs, βCD can load DOX into the hydrophobic cavity via host–guest chemistry during dialysis. The LC and EE of DL-CNPs were determined as 5.3 ± 0.4% and 26.5 ± 1.8%, respectively. These values were comparable to the βCD-labeled CNP models described in our previous study [25].

The DOX release profile of the DL-CNPs was investigated under different conditions mimicking the tumor microenvironment for 48 h. At 37 °C, DL-CNPs were exposed to different GSH concentrations (10 mM and 2 μM) at different pH (5.5 and 7.4). As shown in Figure 2b, >90% of DOX was rapidly released from the DL-CNPs at pH 5.5 in the presence of the 10 mM GSH concentration, which is a similar environment to that of breast cancer. Interestingly, ~80% of DOX was released under pH 7.4 in the presence of 10 mM GSH, which was significantly higher than that with 2 μM GSH at pH 5.5 (<20%). Indeed, the effect of GSH was greater than that of pH on the release of DOX from DL-CNPs.

Typically, βCDs are directly conjugated on the surface of nanoparticles and the drug is loaded in βCD. The drugs are partly exposed to the outer environment so external triggers can easily interfere with the drug-βCD interaction, causing drug release. This conventional βCD-based drug delivery platform exhibited a significantly slow drug release rate at low pH [37,38,39,40]. However, the drug release of DL-CNPs was not the same as other βCD-possessing nanoparticles due to its unique structure. To explain the release kinetics of DL-CNPs, two main factors were considered: steric hindrance and host–guest chemistry. In this research, the surface of the DL-CNPs was modified with two different blocks: a block consisting of SS-βCD branches for drug loading and another block consisting of SS-PEG-SS-HER for PEGylation. Compared to the SS-βCD branches, PEGylated HER branches (SS-PEG-SS-HER) were long, bulky, hydrophilic, and flexible. In an aqueous environment, SS-PEG-SS-HER chains surround the small SS-βCD blocks on the surface of CNPs to improve the solubility of DL-CNPs and the protection of DOX in βCD from premature release in blood vessels [41,42,43]. When the DL-CNPs are delivered to the tumor, they are degraded in response to the high concentration of GSH, and DOX in βCD is released with exposure to an acidic environment. In order to release more DOX from DL-CNPs, steric-hindered SS-PEG-SS-HER chains should be degraded to dissociate PEGylation layer and host–guest chemistry is disrupted by external stimuli. Due to the unique characteristics of a GSH-responsive degradation system with disulfides, our platform exhibited slow and low drug release kinetics of DL-CNPs in the presence of 2 µM GSH at pH 5.5, compared to 10 mM GSH at pH 7.4.

The size of the degraded DL-CNPs was characterized using DLS (Figure 2a). DS-CNPs were found to have a stable and monomodal distribution with an average diameter of ~245 nm, whereas large aggregates formed after the degradation of DL-CNPs. This result supports the destabilization of DL-CNPs after cleavage of disulfide linkages and the release of DOX in a rapid and controlled manner.

### 3.3. Evaluation of Active Targeting and Intracellular Accumulation In Vitro

The selective targeting properties of anticancer drug carriers are key in enhancing their anticancer efficacies and reducing side effects. The significant difference in HER2 levels between HER2-negative and HER2-positive breast cancer enables HER-containing drug carriers to access HER2-positive breast cancer [44,45,46]. Prior to in vitro studies, validation of HER2 expression in the SK-BR-3 and MDA-MB-231 cell lines was conducted with SDS-PAGE and immunoblot analysis. Membranes were incubated with antibodies against HER2 and β-actin for standardization to control the loading variability. As shown in Figure S1, SK-BR-3 expresses a greater amount of HER2 compared to MDA-MB-231 and these results suggest that the difference in cell viability could be attributed to the abundance of HER2 receptors that changes the fate of nanoparticles [47].

**Figure 3:**
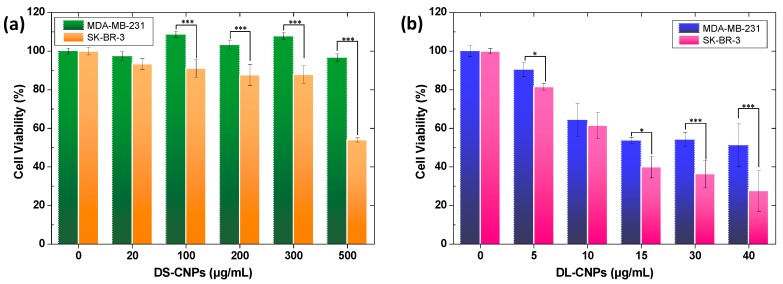
Cytotoxicity of (**a**) DS-CNPs and (**b**) DL-CNPs in SK-BR-3 and MDA-MB-231 cells after 48 h incubation (n = 6, * *p* < 0.05, *** *p* < 0.001).

The in vitro cellular uptake of DS-CNPs in SK-BR-3 (HER2-positive) and MDA-MB-231 (HER2-negative) was evaluated by comparing cell viability after incubation for 48 h. As shown in Figure 3a the viability of SK-BR-3 cells gradually decreased with an increase in the amount of DS-CNPs, whereas the viability of the MDA-MB-231 cells was >96% at up to 500 μg/mL, which suggests that DS-CNPs have low toxicity to HER2-negative cells. These results indicate that DS-NPs have an excellent active targeting ability toward HER2-positive breast cancer, but a high dose of DS-NPs is required to achieve the desired cytotoxicity. Many clinical trials have reported that treatment of HER alone has a low response rate against the extracellular domain of HER-2-positive breast cancer [48,49,50,51]. On the other hand, a combination therapy with chemotherapeutic drugs promotes not only the inhibition of tumor growth, but also the overall survival rate of breast cancer patients [52,53,54,55,56]. To determine the synergistic effect of combination therapy with HER and DOX, the viability of DL-CNPs in SK-BR-3 cells was compared with that in MDA-MB-231 cells. Figure 3b shows significantly decreased cell viability values of SK-BR-3 compared with the viability of MDA-MB-231 incubated at the same DL-CNPs concentration.

**Figure 4:**
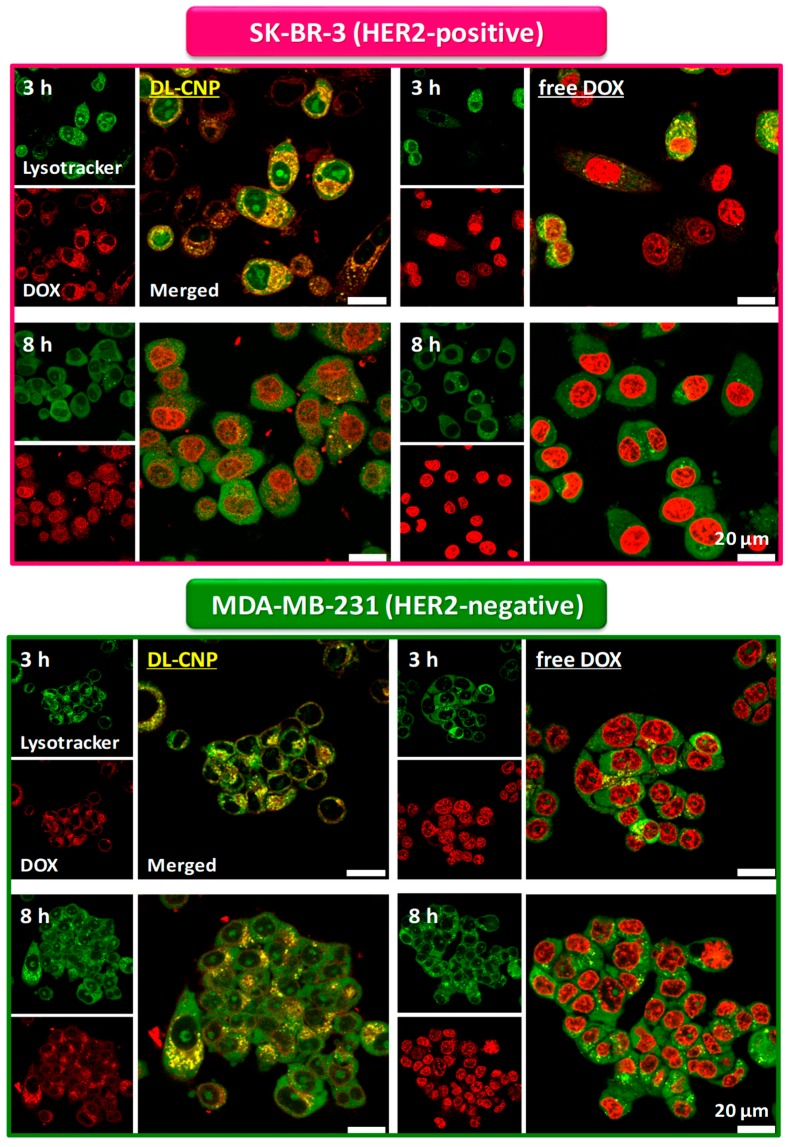
Confocal laser scanning microscopy (CLSM) images (scale bar = 20 μm) of SK-BR-3 and MDA-MB-231 cells incubated with DL-CNPs and free DOX for 3 and 8 h.

To determine the uptake specificity and sequential release of DOX, the DL-NPs and free DOX were incubated in SK-BR-3 and MDA-MB-231 cells, respectively, and the intracellular uptake and localization were investigated by CLSM at different time points (3 h and 8 h). As seen in Figure 4, the red DOX fluorescence intensity percentage was quantified by the ratio of the nucleus to the whole cell area. For comparison, the amount of free DOX was adjusted to be equivalent to the amount of DOX encapsulated in DL-CNPs. Strong intensity in the CLSM images was expected since DOX internalizes into the cells via passive diffusion. After 3 h of incubation, a strong red-color signal corresponding to DOX entrapped in DL-CNPs was widely distributed in the cytoplasm of SK-BR-3, whereas a weak red fluorescence was evident in MDA-MB-231. Similar fluorescence intensity of the DOX was observed in the nucleus of SK-BR-3 and MDA-MB-231. After 8 h, however, the DOX signal in the nucleus had increased by over five times in SK-BR-3 (results were statistically significant at *p* < 0.001), while the increase in MDA-MB-231 was not statistically significant. These results show that HER on the surface promoted the uptake specificity of DL-CNPs in HER2-positive breast cancer cells. The increased accumulation of DOX in the SK-BR-3 cells revealed that dual stimuli in cancer cells, low pH, and the high concentration of GSH facilitated the sequential and controlled release of DOX upon DL-CNP degradation. Similar to free DOX, the green fluorescence of the lysotracker indicated that the DOX released from the DL-CNPs was rapidly localized to the nucleus up to 8 h. DOX is known to inhibit cell proliferation by encoding the gene in the nucleus [57]. These results are consistent with the in vitro release profile in Figure 2b and cell viability results shown in Figure 3b above. These remarkable results confirm that DL-CNPs have great potential for use in the treatment of HER2-positive breast cancer.

### 3.4. In Vivo Antitumor Efficacy and Toxicity of DL-CNPs

**Figure 5:**
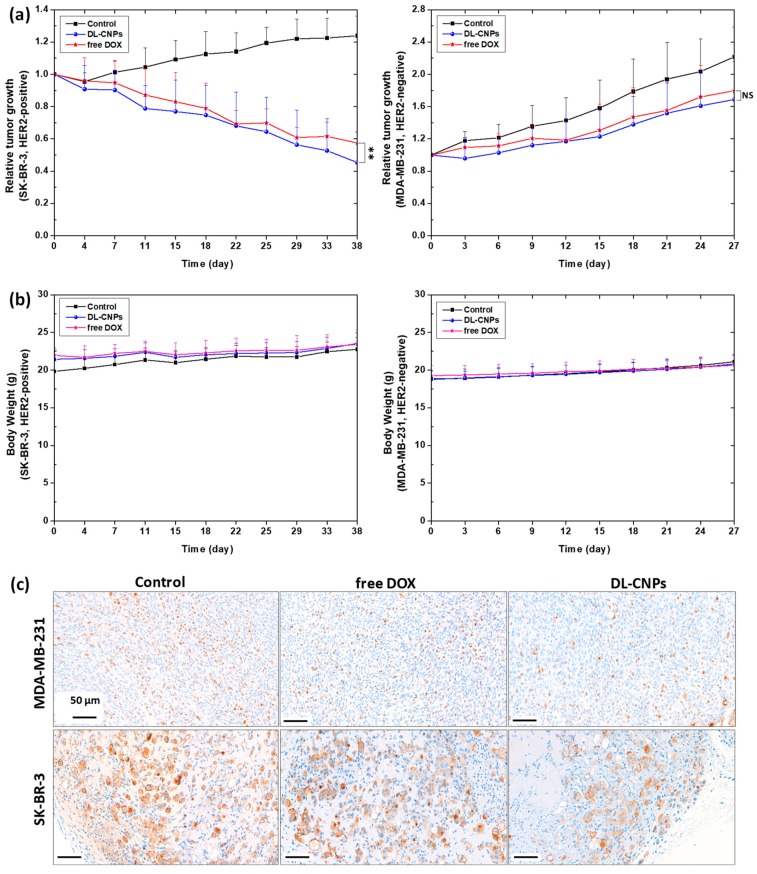
In vivo evaluation of DL-CNPs against HER2-positive and negative xenograft models based on (**a**) tumor growth, (**b**) body weight, and (**c**) histological analysis of Ki-67 expression in representative tumor tissue sections. For drug treatment groups, 1 mg/kg of DOX was injected weekly within 200 μL saline (n ≥ 7 per group).

To evaluate the antitumor efficacy of the DL-CNPs against breast cancer, xenografts implanted with SK-BR-3 and MDA-MB-231 were treated with DL-CNPs, free DOX (an equivalent amount to the DOX in DL-CNPs), and PBS as the control. One mg DOX/kg body weight was weekly injected and the relative tumor growth over time was statistically analyzed by two-way analysis of variance (ANOVA) in GraphPad Prism 5 (GraphPad Software, La Jolla, CA, USA). As shown in Figure 5a, the SK-BR-3 tumor growth of the mice treated with DL-CNPs significantly decreased (*p* < 0.01) compared with that of MDA-MB-231. Furthermore, immunohistochemical analysis using Ki-67 staining in Figure 5c supported that treatment of DL-CNPs exhibited more effective suppression of tumor proliferation than free DOX in the SK-BR-3 groups, which indicates a synergistically enhanced antitumor effect from the dual delivery of HER and DOX in DL-CNPs. However, the body weight was fairly constant, which was comparable to the control group during the treatment period (Figure 5b).

## 4. Conclusions

Multi-functional CNPs engineered with PEG, HER, disulfides, and DOX were introduced as anticancer drug carriers for the treatment of HER2-positive cancer. HER on the surface of the nanoparticles promoted the cellular uptake of DS-CNPs into HER2-positive cells with low toxicity. DL-CNPs are stable under physiological conditions; however, DL-CNPs destabilized after endocytosis upon the cleavage of both disulfides, and acid-labile amide bonds enable the release of DOX in a controlled manner. Treatment with DL-CNPs resulted in the significant inhibition of tumor growth in xenografts derived from SK-BR-3 human breast cancer cells. Furthermore, the combined treatment of HER and DOX promoted an anticancer effect compared to the treatment with DOX alone. These remarkable results support that DL-CNP is an excellent platform for delivering HER2-positive breast cancer therapy.

## Supplementary Materials

The following are available online at https://www.mdpi.com/2079-4991/10/1/91/s1, Figure S1. (a) Expression of HER2 in SK-BR-3 and MDA-BM-231 breast cancer cell lines. β-actin served as loading control; (b) Quantification of HER2 protein expression is given as intensity of β-actin-standardized HER2 levels in the cells.
