# Supplementary material for: Dual pH- and GSH-Responsive Degradable PEGylated Graphene Quantum Dot-Based Nanoparticles for Enhanced HER2-Positive Breast Cancer Therapy
Source: Nanomaterials (Basel). 2020 Jan 2;10(1):91. doi: 10.3390/nano10010091 (PMC7022825; doi:10.3390/nano10010091)
Supplement: Supplementary file 1 [file nanomaterials-10-00091-s001.pdf]

nanomaterials

MDPI

Supporting information

Dual pH- and GSH-Responsive Degradable PEGylated Graphene Quantum Dot-Based Nanoparticles for Enhanced HER2-Positive Breast Cancer Therapy

Na Re Ko [1,2,†], Se Young Van [1,2,†], Sung Hwa Hong [3], Seog-Young Kim [4], Miran Kim [2], Jae Seo Lee [5], Sang Ju Lee [2], Yong-kyu Lee [6], Il Keun Kwon [5] and Seung Jun Oh [2,*]

1 Biomedical Research Center, Asan Institute for Life Sciences, 88 Olympic-ro 43-gil, Songpa-gu, Seoul 05505, Korea; nare.ko@amc.seoul.kr (N.R.K.); sey.van88@gmail.com (S.Y.V.)

2 Department of Nuclear Medicine, Asan Medical Center, University of Ulsan College of Medicine, 88 Olympic-ro 43-gil, Songpa-gu, Seoul 05505, Korea; ranaranmi@naver.com (M.K.); atlas425@amc.seoul.kr (S.J.L.)

3 Department of Chemical Engineering and Applied Chemistry, University of Toronto, 200 College Street, Toronto, ON M5S 3E5, Canada; sunghwa.hong@mail.utoronto.ca (S.H.H.)

4 Department of Convergence Medicine, University of Ulsan College of Medicine, 88 Olympic-ro 43-gil, Songpa-gu, Seoul 05505, Korea; ksy0222@gmail.com (S.-Y.K.)

5 Department of Dental Materials, School of Dentistry, Kyung Hee University, 26 Kyungheedae-ro, Dongdaemun-gu, Seoul 02447, Korea; leejs8433214@naver.com (J.S.L.); kwoni@khu.ac.kr (I.K.K.)

6 Department of Chemical & Biological Engineering, Korea National University of Transportation, 50 Daehak-ro, Chungcheongbuk-do, Cheongju 27469, Korea; leeyk@ut.ac.kr (Y.-k.L.)

* Correspondence: sjoh@amc.seoul.kr; Tel.: +82-2-3010-4595

† These authors contributed equally to this work.

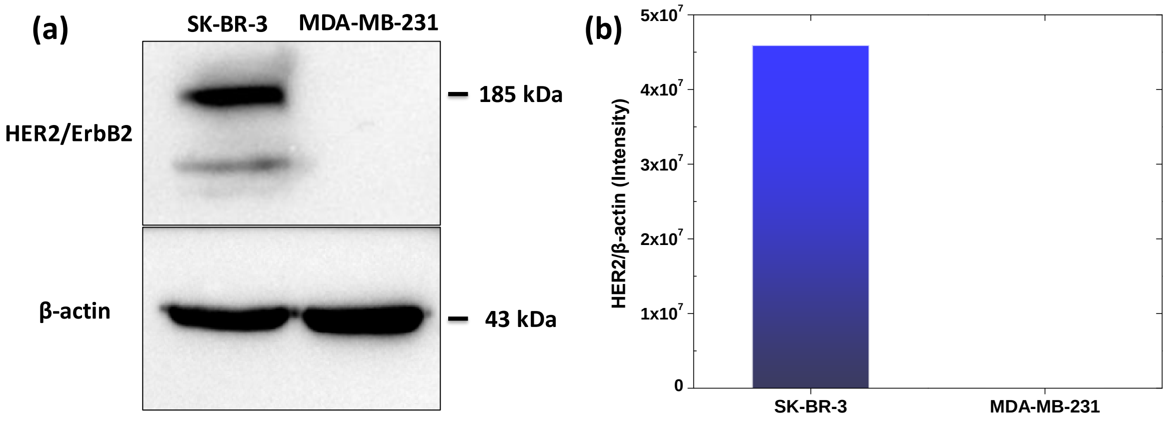

Figure S1. (a) expression of HER2 in SK-BR-3 and MDA-BM-231 breast cancer cell lines. β-actin served as a loading control. (b) Quantification of HER2 protein expression is given as intensity of β-actin-standardized HER2 levels in the cells.
